# Nuclear Exportin Receptor CAS Regulates the NPI-1–Mediated Nuclear Import of HIV-1 Vpr

**DOI:** 10.1371/journal.pone.0027815

**Published:** 2011-11-16

**Authors:** Eri Takeda, Tomoyuki Murakami, Go Matsuda, Hironobu Murakami, Tamotsu Zako, Mizuo Maeda, Yoko Aida

**Affiliations:** 1 Viral Infectious Diseases Unit, RIKEN, Hirosawa, Wako, Saitama, Japan; 2 Laboratory of Viral Infectious Diseases, Department of Medical Genome Sciences, Graduate School of Frontier Science, The University of Tokyo, Wako, Saitama, Japan; 3 Japan Foundation for AIDS Prevention, Chiyoda-ku, Tokyo, Japan; 4 Bioengineering Laboratory, RIKEN, Hirosawa, Wako, Saitama, Japan; University Hospital Zurich, Switzerland

## Abstract

Vpr, an accessory protein of human immunodeficiency virus type 1, is a multifunctional protein that plays an important role in viral replication. We have previously shown that the region between residues 17 and 74 of Vpr (Vpr_N17C74_) contained a bona fide nuclear localization signal and it is targeted Vpr_N17C74_ to the nuclear envelope and then imported into the nucleus by importin α (Impα) alone. The interaction between Impα and Vpr is important not only for the nuclear import of Vpr but also for HIV-1 replication in macrophages; however, it was unclear whether full-length Vpr enters the nucleus in a manner similar to Vpr_N17C74_. This study investigated the nuclear import of full-length Vpr using the three typical Impα isoforms, Rch1, Qip1 and NPI-1, and revealed that full-length Vpr is selectively imported by NPI-1, but not Rch1 and Qip1, after it makes contact with the perinuclear region in digitonin-permeabilized cells. A binding assay using the three Impα isoforms showed that Vpr bound preferentially to the ninth armadillo repeat (ARM) region (which is also essential for the binding of CAS, the export receptor for Impα) in all three isoforms. Comparison of biochemical binding affinities between Vpr and the Impα isoforms using surface plasmon resonance analysis demonstrated almost identical values for the binding of Vpr to the full-length isoforms and to their C-terminal domains. By contrast, the data showed that, in the presence of CAS, Vpr was released from the Vpr/NPI-1 complex but was not released from Rch1 or Qip1. Finally, the NPI-1–mediated nuclear import of Vpr was greatly reduced in semi-intact CAS knocked-down cells and was recovered by the addition of exogenous CAS. This report is the first to show the requirement for and the regulation of CAS in the functioning of the Vpr-Impα complex.

## Introduction

Molecular trafficking between the nucleus and the cytoplasm is tightly regulated in eukaryotic cells. Nuclear import processes involve the nuclear pore complexes (NPCs) of the nuclear envelope and, typically, require nuclear localization signals (NLSs). The nuclear import of classical NLS-bearing proteins is mediated by specific soluble factors, including Importin (Imp), which consists of two subunits, Impα and Impβ, small GTPase Ran/TC4, and nuclear transport factor 2 [1]. The ternary complex with NLS-bearing protein, Impα, and Impβ translocates into the nucleus, and the binding GTP-bound form of Ran to Impβ triggers the dissociation of ternary complex, releasing Impα [2]. However, there are many additional pathways that mediate nuclear import; for example, Impβ-like molecules (such as the transport factor for substrates carrying the M9 shuttling signal or importin 7) and Impβ itself are competent to transfer some cargo by themselves [3]. In addition, it was previously reported that Impα could migrate into the nucleus in an Impβ- and Ran-independent manner [4]. Impα alone can escort Vpr, one of the accessory proteins of human immunodeficiency virus type 1 (HIV-1) [5], [6], as well as Ca^2+^/calmodulin-dependent protein kinase type IV (CaMKIV) into the nucleus without utilizing the classical Impβ-dependent transport system [7].

Impα is composed of a flexible N-terminal Impβ-binding (IBB) domain and a highly structured domain comprising ten tandem armadillo (ARM) repeats [2]. The helical ARM repeats assemble into a twisted slug-like structure whose belly serves as the NLS-binding groove. The central portion of Impα, which contains the ARM repeats, recognizes the NLS cargo, while its N-terminal basic region, termed the IBB domain, binds to Impβ, and the region between residues 383 and 497, corresponding to the ninth and tenth ARM regions, binds to the cellular apoptosis susceptibility (CAS) protein [2], [8]. The crystal structure of Impα has shown that the region between residues 469 and 478, within the tenth ARM region, contains the core sequences for CAS binding [8]. The nature of the dissociation of the NLS cargo from Impα is unclear, but it has been proposed that nucleoporins (Nups), together with CAS, assist in the dissociation process [2], [9]. CAS binds preferentially to Impα, after its dissociation from the NLS cargo, and exports nuclear Impα to the cytoplasm. However, Impα has at least seven isoforms in human [2], [10], [11], grouped into three subfamilies (α1, α2 and α3) based on their amino acid sequence similarities. There is approximately 80–90% sequence homology in each subfamily [2], [12]. Subfamily α1 includes importin α5 (NPI-1/SPR1/karyopherin alpha 1 [KPNA1]), importin α6 (KPNA5) and importin α7 (KPNA6). Subfamily α2 contains importin α1 (Rch1/SRP1α/KPNA2) and the recently-reported importin α8 (KPNA7) [10], [11]. Subfamily α3 includes importin α3 (Qip1/SRP3/KPNA4) and importin α4 (KPNA3). Members of the three subfamilies have about 50% homology with each other [2], [12]. Many studies have shown that Impα isoforms differ in their efficiencies with respect to classical substrate-specific import, show unique expression patterns in various tissues and cells, and depend on the state of cellular metabolism and differentiation [2], [6], [13]. Taken together, this information suggests that Impα proteins contribute primarily to tissue-specific nuclear transport.

Vpr has multiple biological functions, including nuclear localization activity [14], [15], [16], arresting cells at the G2/M phase of the cell cycle [17], [18], [19], increasing the activity of the HIV-1 long terminal repeat [20], selective inhibition of cellular pre-mRNA splicing both *in vivo* and *in vitro* [21], [22], and positive and negative regulation of apoptosis [23]. These functions are carried out through interactions with a variety of cellular partners. Especially, the virion-associated viral protein, Vpr, is necessary for the nuclear import of the viral pre-integration complex (PIC) in non-dividing cells [6], [14], [15], [24], although its exact role in the PIC entry mechanism remains unclear. There are several pathways that Vpr could use to cross the nuclear envelope. First, numerous investigations regarding the subcellular localization of Vpr suggest that the Vpr protein may cross the nuclear envelope by passive diffusion, as it is small enough (15 kDa) to pass through the NPC [15], [25]. Second, Vpr enters the nucleus by interacting with nucleoporins, which are constituents of the NPC [26], [27], [28]. Third, Vpr binds to Impα , which stimulates subsequent nuclear import of the cargo by increasing the affinity of Impα for NLS-containing proteins [16]. Another report describes a novel nuclear import mechanism for Vpr, involving two putative alpha-helical domains, located between residues 17 and 34 (αH1) and between residues 46 and 74 (αH3), which are required for the nuclear localization of Vpr [25]. A subsequent study used microinjection and *in vitro* transport assays incorporating the chimeric protein Vpr_N17C74_ to show that the entire region between residues 17 and 74 is a bona fide NLS [5]. Furthermore, an *in vitro* transport assay experiment designed to identify the factors required for Vpr_N17C74_ nuclear entry found that Vpr itself is targeted to the nuclear envelope and is then transported by Impα, without any involvement of Impβ [5]. The three typical Impα isoforms, Rch1, Qip1 and NPI-1, appear able to interact directly with Vpr_N17C74_ and support its nuclear entry. Interestingly, the interaction between Impα and Vpr is necessary not only for the nuclear import of Vpr but also for HIV-1 replication in macrophages [6]. These results suggest that the interaction between Vpr and Impα may be a potential target for therapeutic intervention. Indeed, a potential parent compound, hematoxylin, has been identified, which suppresses the Vpr_N17C74_-Impα interaction, thereby inhibiting the nuclear import of the HIV-1 viral genome in macrophages in a Vpr-dependent manner [29].

A nuclear magnetic resonance structural analysis revealed that full-length Vpr forms three amphipathic alpha helices surrounding a hydrophobic core [30], [31]. It has a flexible, negatively-charged N-terminal domain flanking the helices and its C-terminal domain is also flexible, positively charged, and rich in arginine residues [30], [31]. Two motifs, amino acids 56 to 77 in the third α-helical domain (αH3) and amino acids 77 to 96 in the arginine-rich C-terminal domain, are critical for the inhibition of pre-mRNA splicing by Vpr [20], while the C-terminal domain appears to be critical for Vpr-induced G2 arrest and apoptosis [32], [33]. The N-terminal domain was shown to be important for localization to the nuclear rim [34]. Taken together, these results clearly indicate that the N-terminal and the C-terminal Vpr domains play critical roles in the multiple functions of Vpr. However, it is unclear whether full-length Vpr enters the nucleus in a manner similar to that of the chimeric protein, Vpr_N17C74_. In this investigation, we have studied the detailed mechanism of full-length Vpr entry into the nucleus. Using a digitonin-permeabilized transport assay, the nuclear import of full-length Vpr by the three major isoforms of Impα, Rch1, Qip1 and NPI-1, was analyzed. Furthermore, to clarify the means by which NPI-1 selectively transports full-length Vpr, the Impα isoform domain involved in the interaction with Vpr and these accurate binding affinities were identified using a glutathione-S-transferase (GST)-pull down assay and surface plasmon resonance (SPR). Moreover, we used a GST pull-down assay to show that although Vpr binds to the CAS-binding domain of all of three Impα isoforms to roughly the same extent, CAS can dissociate the interaction between Vpr and NPI-1 but not between Vpr and Rch1 or Qip1. Finally, we used an *in vitro* nuclear import assay using HeLa cells with knocked-down CAS to demonstrate that CAS is required for the nuclear entry of full-length Vpr.

## Results

### Full-length Vpr is preferentially imported into nuclei by Impα5 (NPI-1)

**Figure 1:**
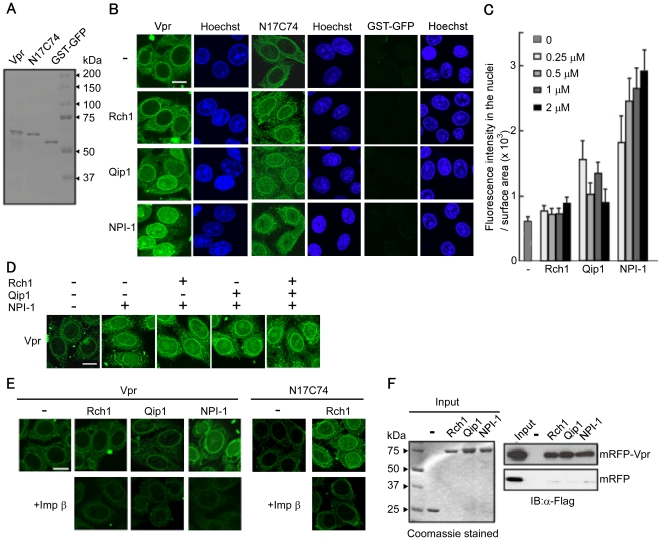
Importin α5/NPI-1 preferentially mediates the nuclear import of Vpr. (A) Twenty-five pmol of purified recombinant GST- and GFP-tagged Vpr (Vpr), GST- and GFP- tagged Vpr_N17C74_ (N17C74), GST-tagged GFP (GST-GFP) were resolved by 10% SDS-PAGE and stained with Coomassie brilliant blue (CBB). (B) Nuclear import of Vpr by importin α (Impα) isoforms. Digitonin-permeabilized HeLa cells were incubated with 1 µM of Vpr, N17C74, and GST-GFP in the absence (-) or presence of 1 µM (for Vpr and GST-GFP) or 3 µM (for N17C74) of each of the recombinant Impα isoforms, Rch1, Qip1 and NPI-1. Cells were fixed in 3.7% formaldehyde and stained with Hoechst 33342 to show the position of the nucleus (right panel). After fixation, cells were analyzed by confocal laser scanning microscopy. Bar = 10 µm. (C) Fluorescence intensity of Vpr per surface area was quantified for at least 70 nuclei in the presence of the indicated concentrations of the Impα isoforms from three independent experiments. The bar shows the standard errors of measurements. (D) *In vitro* nuclear import assay for GST-GFP-Vpr was performed in the absence (-) or presence of 1 µM of the Impα isoforms. After fixation, cells were analyzed by confocal microscopy. Bar = 10 µm. (E) *In vitro* nuclear import assay for Vpr was performed in the absence (-) or presence of 1 µM of the Impα isoforms, and 1 µM of Impα isoforms with 1 µM Impβ. N17C74, as a control, was performed with 1 µM of Rch1 and 1 µM Impβ. After fixation, cells were analyzed by confocal microscopy. Bar = 10 µm. (F) Binding assay between Vpr and the Impα isoforms. Glutathione-Sepharose beads were coupled with the GST-Impα isoforms, Rch1, Qip1 and NPI-1 or GST alone, and were incubated with Vpr protein purified from 293T cells transfected with pCAGGS mammalian vectors encoding Flag-mRFP (mRFP), or Flag-mRFP-Flag-Vpr (mRFP-Vpr). The bound fractions and 1/20 of the input of mRFP-Vpr and mRFP were analyzed by immunoblotting with an anti-Flag M2 monoclonal antibody (MAb) (right panel). Twenty-five pmol of GST or GST-Impα isoforms were resolved by 10% SDS-PAGE and stained with CBB (left panel). The positions of mRFP and mRFP-Vpr are indicated.

A chimeric protein comprising full-length Vpr fused at the N-terminus to GST and green fluorescent protein (GFP) (∼63 kDa) was constructed, which surpassed the limit for passive diffusion into the nucleus (**Fig. 1A**). An *in vitro* nuclear import assay was then performed using digitonin-permeabilized, semi-intact HeLa cells (**Fig. 1B**). In the absence of soluble factors, full-length Vpr localized predominantly to the perinuclear region in a manner similar to that of the Vpr_N17C74_ mutant. By contrast, no signal was detected in the perinuclear region when using a negative control protein (a chimeric GST-GFP protein). Interestingly, the nuclear import of Vpr changed significantly in the presence of the different Impα isoforms. High levels of Vpr entered the nucleus in the presence of NPI-1; however, the levels were much lower in the presence of Qip1, and no entry was observed in the presence of Rch1. By contrast, in agreement with a previous report [6], the Vpr_N17C74_ mutant entered the nucleus at similar levels in the presence of all three Impα isoforms. GST-GFP failed to enter the nucleus, even in the presence of all three Impα isoforms.

Next, the extent of the nuclear import activity exhibited by Vpr in the presence of 0.25, 0.5, 1 or 2 µM of the Impα isoforms was examined by measuring the fluorescence intensity in the nucleus (**Fig. 1C** **)**. Only NPI-1 efficiently enhanced the nuclear import of Vpr. Qip1 showed a very weak effect on the nuclear entry of Vpr, which remained at a low level even in the presence of 2 µM Qip1. By contrast, no nuclear import of Vpr was detected in the presence of Rch1, even at a concentration of 2 µM.

The effect of Rch1 and Qip1 on the nuclear entry of full-length Vpr mediated by NPI-1 was then examined (**Fig. 1D** **)**. NPI-1–mediated nuclear import of Vpr did not decrease in the presence of Rch1 or Qip1. Moreover, the Impα isoform-driven nuclear import of Vpr was completely inhibited when Impβ was added to semi-intact HeLa cells (**Fig. 1E**). Likewise, Impβ decreased the nuclear import of the Vpr_N17C74_ mutant in the presence of Rch1 (**Fig. 1E**). Taken together, these results suggest that full-length Vpr is targeted to the perinuclear region and is then transported into the nucleus by NPI-1 alone, without any requirement for Impβ.

### Full-length Vpr interacts with all three Impα isoforms

To examine further whether full-length Vpr interacts directly with all three Impα isoforms, the recombinant GST-tagged Impα isoforms, Rch1, Qip1 and NPI-1 (immobilized on glutathione-Sepharose beads), were incubated with mRFP-Vpr purified from vertebrate cells. Interestingly, full-length Vpr was able to interact with all three isoforms (**Fig. 1F**), indicating that Vpr is able to bind directly to Rch1 and Qip1, even though these isoforms did not promote its nuclear entry as well as did NPI-1, which showed preferential transport of Vpr into the nucleus.

### Full-length Vpr binds to the Impα CAS-binding domain

**Figure 2:**
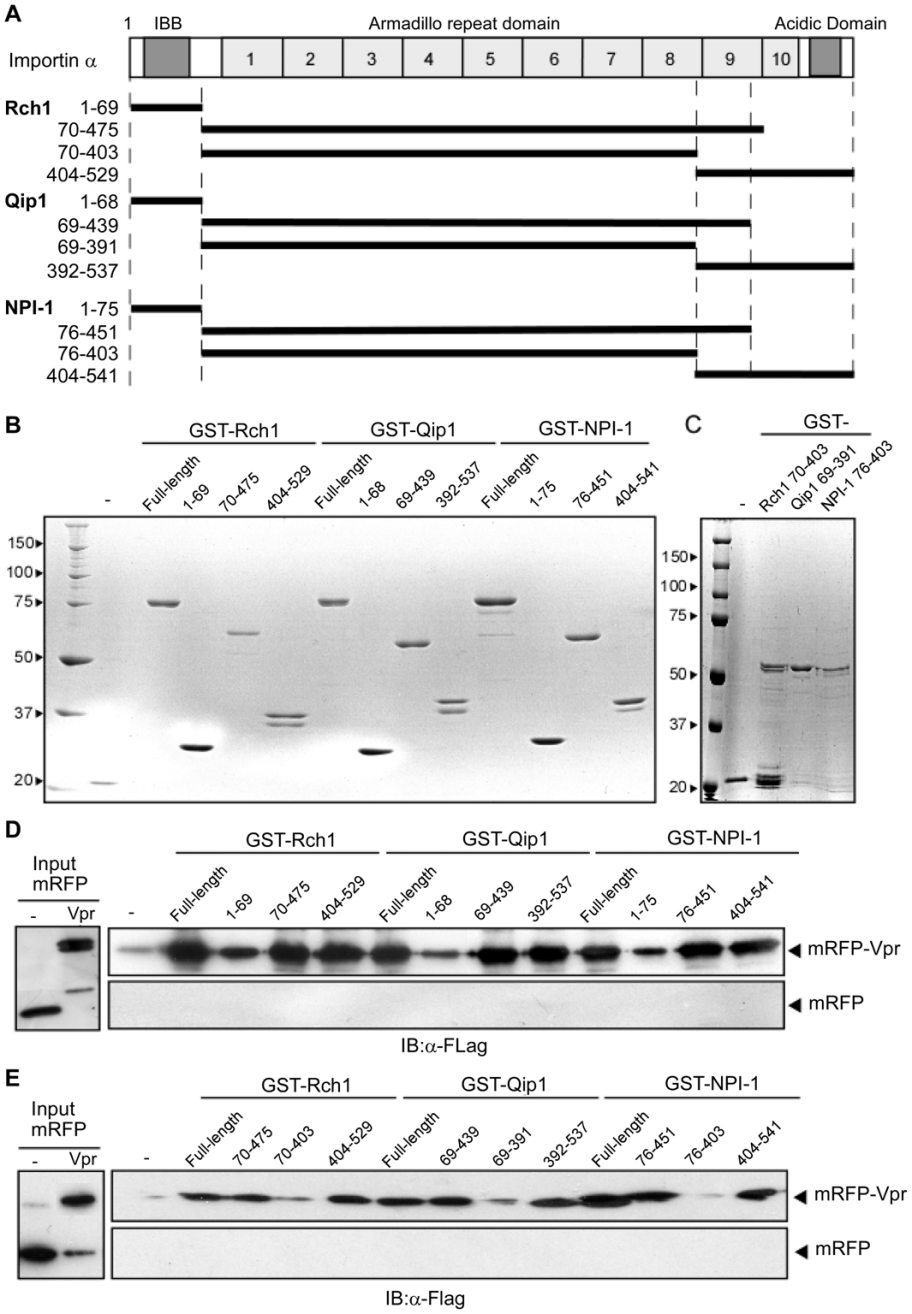
Mapping of the Impα isoform domains involved in the interaction with Vpr . (A) Schematic representation of the Impα isoforms, Rch1, Qip1, NPI-1 and their deletion mutants. (B and C) All mutants were expressed as GST fusion proteins in *E. coli* and purified using Glutathione-Sepharose. Twelve pmol of purified GST-tagged Impα isoform derivatives were resolved by 10% SDS-PAGE and stained with CBB. (D and E) Binding of Impα isoforms to Vpr. Glutathione-Sepharose beads coupled to GST-Impα isoforms or GST alone were incubated with mRFP-Vpr or mRFP. The bound fractions and 1/50 of the input of mRFP-Vpr and mRFP were analyzed by immunoblotting with anti-Flag M2 MAb (right panel). The positions of mRFP and mRFP-Vpr are indicated.

Since the three major Impα isoforms, Rch1, Qip1 and NPI-1, share approximately 50% overall amino acid sequence similarity [2], [12], we decided to determine whether the same domain was involved in binding full-length Vpr in all three isoforms. Impα is composed of an N-terminal IBB domain, a highly-structured domain comprised of ten tandem ARM repeats and a C-terminal acidic domain [2], as shown in **Fig. 2A**. For each isoform, three truncated mutants were prepared as fusion proteins with GST: 1) the IBB domain mutant, 2) the mutant containing the ARM repeat domain but lacking the tenth ARM repeat, and 3) the mutant including the C-terminal region between the ninth ARM repeat and the acidic domain, (**Fig. 2B**). These mutants were then assessed for their binding activity with full-length Vpr (**Fig. 2D**). The recombinant mutant corresponding to the ARM repeat domain between residues 70 to 438 of Rch1 was very unstable and was difficult to purify; therefore, a slightly extended form of the mutant, between residues 70 to 475 but without the tenth ARM repeat, was used.

Vpr bound to all mutants of all three Impα isoforms: two of the deletion mutants, the ARM repeat domain lacking the tenth ARM repeat (Rch1_70–475_, Qip1_69–439_ and NPI-1_76–451_) and the C-terminal region containing the ninth ARM repeat (Rch1_404–529_, Qip1_392–537_ and NPI-1_76–541_), bound to Vpr with the same level as full-length Impα. The IBB domain mutant (Rch1_1–69_, Qip1_1–68_ and NPI-1_1–75_) also interacted with Vpr, albeit with lower affinities than those shown by the full-length Impα isoforms. These results suggested that the main Vpr binding site is located somewhere between the structural ARM repeats and the C-terminal region but is not found in the IBB domain for all three Impα isoforms.

The two mutant forms that bound strongly to Vpr, as mentioned above, shared the ninth ARM repeat (**Fig. 2A**). Therefore, different truncated forms lacking the ninth ARM repeat (Rch1_70–403_, Qip1_69–391_ and NPI-1_76–403_) were constructed (**Fig. 2C**) and a pull-down assay was performed using mRFP-Vpr (**Fig. 2E**). The binding of the ARM repeat mutants lacking the ninth ARM repeat to Vpr was reduced significantly, indicating that the ninth ARM repeat region of all of three Impα isoforms (Rch1_404–475_, Qip1_392–439_ and NPI-1_404–451_) is the major binding site for full-length Vpr.

### Full-length Vpr binds with similar affinity to the C-terminal domain of the three Impα isoforms

**Figure 3:**
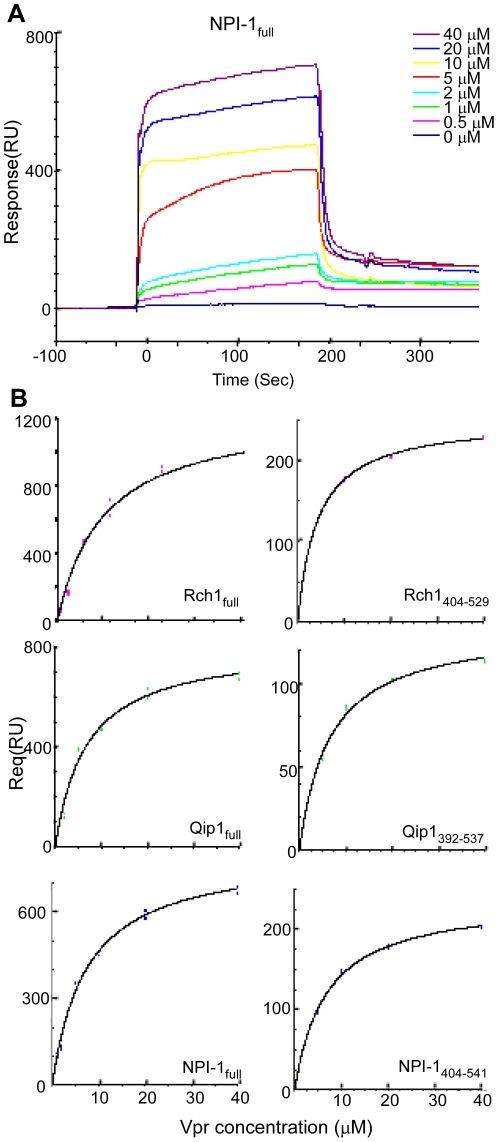
SPR measurements of the interaction between Vpr and full-length or C-terminal Impα isoforms using BIAcore. (A) SPR sensorgrams of the interactions between GST-Vpr and the full-length Impα isoform, NPI-1_full_, immobilized to the CM sensor chip. Sensor curves of the interactions between Vpr at various concentrations (0 to 40 µM) and NPI-1_full_ are shown. (B) The analysis curves used to obtain the dissociation constants (K_D_) for the interactions between Vpr and the Impα isoforms, Rch1_full,_ Qip1_full_, NPI-1_full_, Rch1_404-529_, Qip1_392-537_ and NPI1_404-541_ using the steady state binding model equation (see Material and Methods).

To quantify the binding affinities between Vpr and each of the Impα isoforms accurately, the BIAcore 2000 SPR sensor system was used. In this system, four samples can be immobilized individually on the same chip, and their interactions with analytes can be tested simultaneously. Each of the three recombinant full-length Impα isoforms and their C-terminal peptide mutants (Rch1_404–529_, Qip1_392–537_ and NPI-1_76–541_), the GST was cleaved with PreScission protease, were immobilized on one lane of a sensor chip and a remaining vacant lane was used as a negative control for the non-specific binding of GST-Vpr and GST to the chip. The chip-bound Impα isoforms were exposed to various concentrations of GST-Vpr and GST, and their affinity constants were measured by analyzing the curves (**Fig. 3**). Typical sensor curves of various Vpr concentrations (0 to 40 µM) interacting with full-length NPI-1 (NPI-1_full_) are shown in **Fig. 3A**. The binding affinities obtained are summarized in **Table 1**. The K_D_ values for the full-length Vpr-Impα isoform interactions were very similar: 8.9 µM (Rch1), 6.8 µM (Qip1), and 7.4 µM (NPI-1). The K_D_ values for two of the Vpr-Impα C-terminal peptides were similar to those for the full-length Impα isoforms, 6.5 µM (Qip1_392–537_) and 6.7 µM (NPI-1_404–541_); however, the K_D_ of the Rch1 C-terminal peptide, 4.3 µM (Rch1_404–529_), showed a two-fold decrease compared with the K_D_ of full-length Rch1. This experiment confirmed that the binding affinities between Vpr and all Impα isoforms are very similar.

**Table 1:** Dissociation equilibrium constants determined using BIAcore.

Analite	Ligand	KD (M)	Ligand	KD (M)
Vpr	Rch1_full_	8.9×10^−6^	Rch1_404–529_	4.3×10^−6^
	Qip1_full_	6.8×10^−6^	Qip1_392–537_	6.5×10^−6^
	NPI-1_full_	7.4×10^−6^	NPI-1_404–541_	6.7×10^−6^

Dissociation equilibrium constants determined using BIAcore. K_D_ of Vpr was determined by using the Vpr sensorgrams obtained by subtracting GST sensorgrams from GST-Vpr sensorgrams at the same concentrations. *Full*: full-length.

### CAS disrupts the interaction between Vpr and NPI-1, but not between Vpr an Rch1 or Qip1

**Figure 4:**
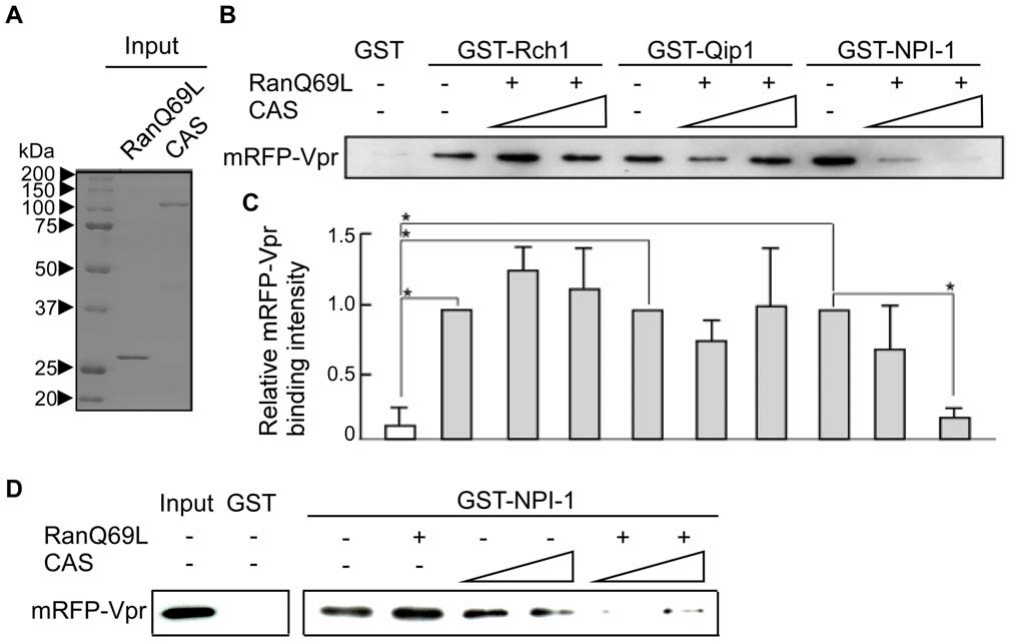
CAS disrupts the interaction between Vpr and NPI-1. (A) Twenty-five pmol of purified recombinant RanQ69L and CAS were resolved by 10% SDS-PAGE and stained with CBB. (B) Glutathione-Sepharose beads coupled with the GST-Impα isoforms, Rch1, Qip1 and NPI-1 (each 25 pmol) or GST (25 pmol), were incubated with mRFP-Vpr, Q69LRanGTP (25 pmol) and/or CAS protein (5 and 50 pmol, respectively). The bound fractions of mRFP-Vpr and mRFP were analyzed by immunoblotting with anti-Flag M2 MAb. (C) The immunoblots of mRFP-Vpr binding were analyzed by densitometry and each sample was normalized to the Impα isoforms without CAS protein. Each column and error bar represents the means ± SD of results from three experiments. The asterisk* represents a *p*-value of <0.0005. (D) Glutathione-Sepharose beads coupled with the NPI-1 (each 25 pmol) or GST (25 pmol), were incubated with mRFP-Vpr, Q69LRanGTP (25 pmol) and/or CAS protein (10 and 50 pmol, respectively). The bound fractions of mRFP-Vpr were analyzed by immunoblotting with anti-Flag M2 MAb. The bound fractions and 1/20 of the input of mRFP-Vpr were analyzed by immunoblotting with anti-Flag M2 MAb. The positions of mRFP-Vpr are indicated.

The sequences required for binding to the CAS nuclear export factor are located between the ninth and tenth ARM repeats within Impα [2]. The present study indicated that the ninth ARM repeat of Impα is the main region involved in binding to Vpr and is also necessary for the interaction with CAS. Therefore, to determine whether CAS affects the interaction between Vpr and Impα, glutathione-sepharose beads coupled to GST-Rch1, -Qip1 or -NPI-1 were incubated with mRFP-Vpr in the absence or presence of purified recombinant CAS and a RanGTP analog (Q69LRanGTP) (**Fig. 4A**). RanGTP is necessary for the interaction between Impα and CAS in cell nuclei. As shown in **Fig. 4B and C**, the amount of Vpr bound to NPI-1 decreased as the concentration of CAS increased in the presence of Q69LRanGTP in a dose-dependent manner (a 0.2-fold difference in the presence of 50 pmoles CAS). This was not the case for Rch1 and Qip1, indicating that CAS causes the dissociation of Vpr from NPI-1 (which can import the full-length Vpr into the nucleus) but does not disrupt Vpr/Rch1 or Vpr/Qip1 interactions, which are not involved in Vpr nuclear import. When Q69LRanGTP was absence on Pull-down assay, CAS only showed a very weak effect on the dissociation of Vpr from NPI-1 (**Fig. 4D**).

### CAS regulates the NPI-1–mediated nuclear entry of full-length Vpr

**Figure 5:**
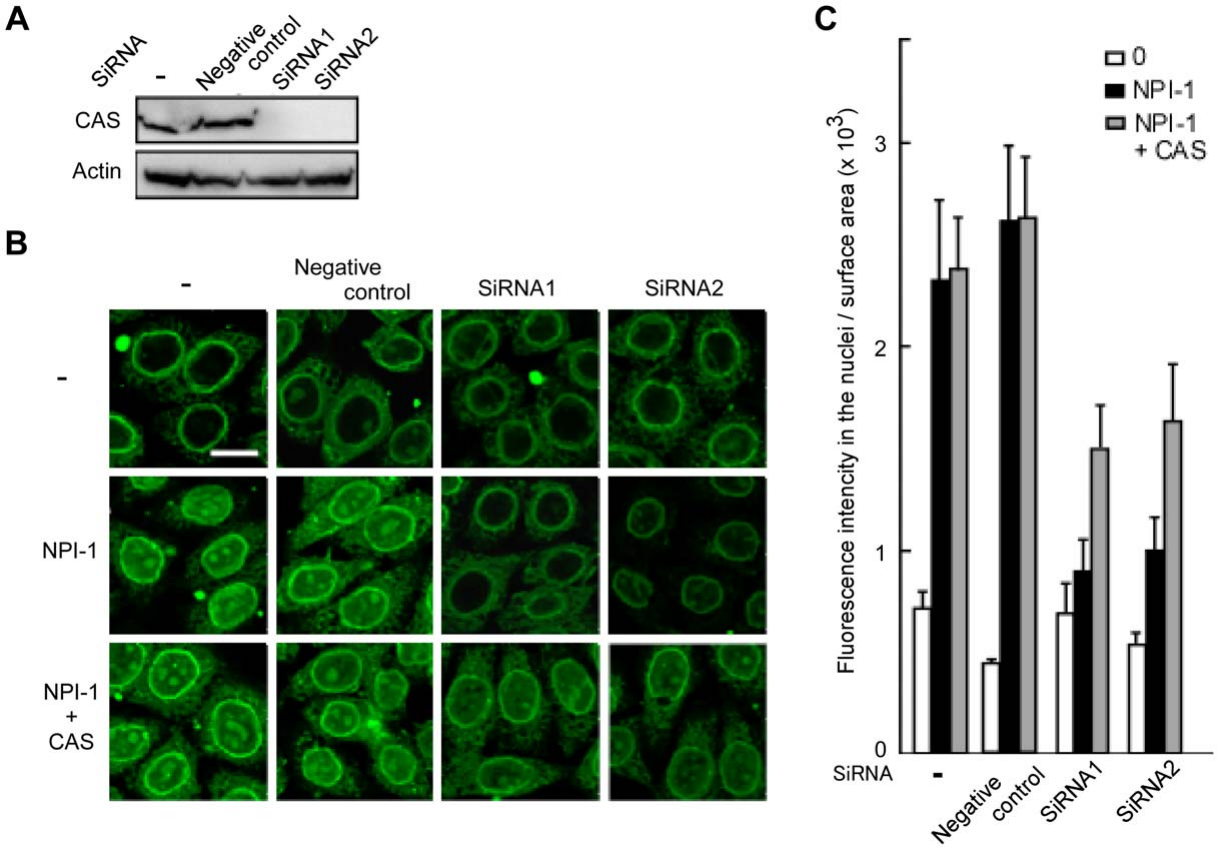
The siRNA-induced knock down of CAS prevents the nuclear import of Vpr. (A) CAS-specific siRNA and nonspecific siRNA (negative control) transfections were performed in HeLa cells. After 36 h of treatment with CAS-specific siRNA1 and siRNA2 and the negative control siRNA, cell extracts were prepared and immunoblotting with an anti-CAS antibody was used to determine the transfection efficiencies of the siRNAs. Untreated HeLa cells (-). Actin was included as an internal control. The positions of CAS and actin are indicated. (B) Digitonin-permeabilized HeLa cells, transfected with siRNAs, were incubated with 1 µM GST-GFP-Vpr, 1 µM NPI-1 and 1 µM CAS. After fixation, cells were analyzed by confocal laser scanning microscopy. Bar = 10 µm. (C) Fluorescence intensity per nuclear surface area was quantified in over 80 nuclei from three independent experiments. The bar shows the standard errors of measurements.

Finally, the requirement for CAS for NPI-1–mediated nuclear entry of full-length Vpr was confirmed using an *in vitro* nuclear import assay. The results clearly showed that the expression of the endogenous CAS protein was not affected by digitonin-induced permeabilization (**Fig. S1**). Therefore, an *in vitro* nuclear import assay was performed using HeLa cells in which CAS expression had been knocked down. Knock-down was confirmed by immunoblotting experiments conducted after a 36 h treatment with two siRNAs (siRNA1 and siRNA2) against CAS mRNA (**Fig. 5A**). HeLa cells were permeabilized with digitonin and used in an *in vitro* import assay (**Fig. 5B, C**). The nuclear import of GST-GFP-Vpr, which was enhanced by the addition of NPI-1, was greatly decreased in HeLa cells treated with either CAS-specific siRNA1 or siRNA2, but not in negative control siRNA-transfected cells or in untreated cells. Furthermore, this reduction in nuclear import was rescued by up to 50% by the addition of exogenous CAS (recovery was considered to be 50% because exogenous CAS needs time to reach the cell nuclei). However, Vpr was able to localize to the nuclear envelope in these cells, indicating that CAS has no effect on the perinuclear localization of Vpr, an event that does not require both Impα isoforms. These results clearly demonstrate that CAS is essential for the NPI-1–mediated nuclear import of Vpr.

## Discussion

This study investigated the nuclear import of full-length Vpr, the HIV-1 accessory protein, using an *in vitro* nuclear import assay with digitonin-permeabilized HeLa cells and a pull-down assay. The results produced two major conclusions: first, the data suggested that full-length Vpr is preferentially imported into the nucleus by NPI-1 but not Rch1 and Qip1, in contrast with Vpr_N17C74_, which can be imported by all three major isoforms of Impα [6]. Certain previous studies have shown that each Impα isoform imports different viral proteins; for example, Qip1 interacts with HIV-1 integrase (IN) and contributes to HIV-1 nuclear import and replication [35], while NPI-1 and Rch1 interact with the influenza virus Nucleoprotein to promote its nuclear import [36]. Second, our data from the *in vitro* nuclear import assay using HeLa cells with the knocked-down nuclear export receptor, CAS, indicated that CAS is essential for the NPI-1–mediated nuclear import of Vpr. We also showed that CAS mediated the release of Vpr from NPI-1 but not from Rch1 and Qip1, thus facilitating the transport of Vpr into the nucleus. It was known, from previous reports, that in the classical nuclear import of the NLS cargo/Impα/Impβ complex, CAS increased the dissociation of the Impα/NLS cargo complex together with nucleoporins, such as Nup50, after the dissociation of Impβ from the ternary complex in the nucleus [2], [37], [38]. However, a requirement for CAS in this process had not previously been confirmed by *in vitro* nuclear import assay. In addition, it was previously reported that although CaMKIV, which is transported by Impα without utilizing Impβ, binds to the C-terminal region of mouse Rch1 (Rch1_413–459_) in a similar manner to Vpr, the interaction between Impα and CaMKIV was not disrupted by the addition of CAS in a solution-binding assay [7]. Therefore, this study is the first to demonstrate, using *in vitro* nuclear import and pull-down assays, that CAS is required for Impα-mediated nuclear import and plays a direct role in the regulation of the NLS cargo-Impα complex without utilizing the Impβ-dependent transport pathway.

Our present and previous results have allowed us to characterize the mechanism governing the entry of full-length Vpr into the nucleus as follows: i) full-length Vpr localizes to the perinuclear region, without a requirement for soluble factors, before it is transported into the nucleus by Impα, as shown by the *in vitro* nuclear import assays using digitonin-permeabilized HeLa cells (**Fig. 1**) and CAS-specific siRNA-treated permeabilized HeLa cells (**Fig. 5**). This perinuclear localization in the absence of Impα isoforms is in agreement with the nuclear import of Vpr_N17C74_ (**Fig. 1B**) [6] and distinguishes the nuclear import of Vpr from that of other NLS-bearing proteins. ii) The detailed binding assay with truncated forms of the three Impα isoforms showed that full-length Vpr binds preferentially to the ninth ARM repeat, which is also the domain required for CAS interaction with Impα. This data partially agrees with a previous report in which Vpr_N17C74_ required the C-terminal peptide of Impα directly to entry into nucleus, though it majorly bound to IBB domain of the Impα [5]. iii) Our SPR analysis clearly demonstrated similar binding affinities for Vpr to each of the three full-length Impα isoforms as well as to their C-terminal domains, which contained the ninth ARM region, identified as the major Vpr-binding site, and also the CAS binding site [8], [37]. iv) This study demonstrated that the release of Vpr from the Vpr/NPI-1 complex depends on CAS. By contrast, CAS did not cause the dissociation of Vpr from complexes with Rch1 or Qip1, even though they were capable of importing Vpr into the nucleus (**Fig. 4B, C**). v) We also showed that the nuclear import of Vpr by NPI-1 was not affected by Rch1 or Qip1 (**Fig. 1D**), suggesting that each of the Impα isoforms exist in equilibrium with Vpr in the cytoplasm. It was assumed that all the Impα isoforms have same binding affinity for Vpr (**Fig. 3** and **Table 1**). vi) After interacting with Impα at the perinuclear region, full-length Vpr was selectively imported by NPI-1 but not by Rch1 or Qip1, in contrast to the import of Vpr_N17C74_ by all three isoforms of Impα (**Fig. 1B** **)** [6]. In addition, the NPI-1–driven nuclear import of Vpr appeared to be completely inhibited when Impβ was added to the *in vitro* import assay as shown in **Fig. 1E**. Thus, it seems that the transport of full-length Vpr is mediated in an Impα-dependent/Impβ-independent manner, as was found previously for Vpr_N17C74_ [5], [6]. vii) In an *in vitro* nuclear import assay using HeLa cells with knocked-down CAS, we showed that CAS promotes the NPI-1–mediated nuclear import of Vpr. Taken together, the results suggested that the differences in the dissociation rates for the interactions between Vpr and the three Impα isoforms might permit the novel nuclear import of full-length Vpr specifically mediated by NPI-1. Data from the present study leads us to speculate that the Vpr N- or C-terminal region will bind to the ninth ARM region of Impα with the potential regulation of the nuclear import process through the dissociation of Vpr from NPI-1 via an interaction with CAS. Indeed, it has been reported that the C-terminal region of Vpr, which most closely resembles a classical NLS, is highly involved in its nuclear localization [**39]**, [40****].

It is unclear how the selective release of full-length Vpr from NPI-1 depends on CAS; however, there two possible hypotheses with regards to its mechanism: first, it is predicted that since the binding affinities of Vpr for the C-terminal domain were almost the same for all three Impα isoforms, CAS must be attracted to specific amino acids in NPI-1. Interestingly, the alignment of the sequences of the ninth ARM motif, which are involved in the binding of Vpr, showed that the three Impα isoforms share only 50% overall amino acid sequence similarity [2], [11], [12], suggesting that the ARM motif of NPI-1 may be more effective at binding CAS than that of Rch1 or Qip1. The second possibility relates to the targeting of Vpr to the perinuclear region. Sun *et al*. [38] showed that Impα/NLS cargo complexes, without Impβ, dissociated in the presence of CAS and RanGTP at the nuclear pore complexes. They also speculated that Nup50 facilitates the dissociation of Impα/NLS cargo complexes in the presence of CAS and RanGTP when it reaches the nuclear basket region of the NPC [38]. In a recent report, Ogawa *et al.* [9] speculated that the dissociation of Impα from the NLS-substrate was promoted by Npap60 (Nup50). In addition, interactions between transport factors and key nucleoporins, such as Nup1p, Nup2p and Nup50, appeared to accelerate the formation and dissociation of the NLS cargo/Impα/Impβ complexes [38]. Likewise, in this study, we have also shown that the dissociation of the Vpr/NPI-1complexes may occur at the perinuclear region using an *in vitro* nuclear import assay with digitonin-permeabilized HeLa cells. In this assay, full-length Vpr was targeted directly to the perinuclear region in the absence of soluble factors, and, in addition, this perinuclear localization increased in a dose-dependent manner upon the addition of NPI-1. Earlier studies confirmed that Vpr can interact with nuclear pore complex components [15], [16], [27], [41] and we have previously demonstrated that the interaction between Vpr and the NPC is crucial for Vpr nuclear import, since Vpr mutants, with barely detectable perinuclear localization, could not be imported into the nucleus [5]. Further studies on the role of Vpr at the NPC are now essential for a full understanding of the mechanism of CAS-regulated, NPI-1–mediated nuclear import of full-length Vpr.

Our results clearly indicate that the ninth ARM repeat region of all of three Impα isoforms is the major binding site for full-length Vpr. In contrast, we here demonstrate that the IBB domain of Impα interacts with full-length Vpr, albeit with lower affinity than those shown by the full-length Impα isoforms to their C-terminal domains. This result partially corresponds to our previous finding that Impα binds strongly to Vpr_N17C74_ via the IBB domain, but this binding is not essential for the nuclear entry of Vpr [5]. The IBB domain contains an NLS-like sequence (49-KRRNV-53) that binds to autologous NLS-binding sites in a similar way to the NLS of SV40. Thus, Impα appears to be prevented from binding to a classical-type NLS by an internal NLS until Impβ binds to the IBB domain [42]. These facts suggest that Vpr might modulate the interaction between a classical NLS-bearing protein and Impα, as does Impβ. Interestingly, Bukrinsky and colleagues [16], [43] reported that Vpr associates with the N-terminal region of Impα, which overlaps with the IBB domain of Impα and differs from the classical NLS cargo binding site. This interaction may stimulate nuclear import of the cargo by increasing the affinity of Impα for NLS-containing proteins, including that of HIV-1 matrix (MA) protein, which is one of the components of the PIC and has a basic type of NLS. Thus, Vpr might accelerate nuclear import of the PIC through interaction with the IBB domain, in addition to the NPI-1-driven nuclear import of Vpr, that requires the C-terminal domain of Impα.

Various factors are reported to adapt Impα isoforms for nuclear import. Viral proteins, such as the herpes virus open reading frame (ORF) 57 protein [44], the Influenza virus nucleoprotein [45], [46], and polymerase PB2 [47], appear to be transported by NPI-1. Likewise, it was recently shown that HIV-1 IN appears to interact with Qip1 and contributes to the nuclear import of PIC and viral replication [35]. The results of the present study show that Vpr is selectively imported into the nucleus by NPI-1, and previous work shows that the interaction between Impα and Vpr is necessary not only for the nuclear import of Vpr but also for HIV-1 replication in macrophages [48]. Macrophages are a major target for HIV-1 and serve as a viral reservoir that releases small amounts of viral particles in symptomatic carriers [49]. A striking feature of HIV-1 is its ability to replicate in non-dividing cells, particularly in macrophages. Replication in non-dividing cells depends on the active nuclear import of the viral PIC, which includes the viral proteins, IN, Vpr, and small amounts of MA, in addition to viral nucleic acids [48]. Vpr is particularly important for the nuclear import of the PIC in non-dividing cells [6], [14], [15], [24], although its exact role in the PIC entry mechanism remains to be clarified. Work is currently ongoing to study the expression of Impβ in human differentiated macrophages, and preliminary data suggest that it is expressed at very low levels in primary differentiated macrophages. The low level of Impβ expression in macrophages may result in the inefficient nuclear import of MA and IN, which utilize the classical Impα/Impβ-dependent nuclear import pathway. By contrast, previous studies show that all three Impα isoforms are strongly expressed at both the mRNA and protein levels [6]. This suggests that, although Vpr utilizes many nuclear import pathways [6], [16], [26], [27], [28], the Impα-mediated nuclear import pathway is the most efficient in macrophages. In summary, the results of the present study show for the first time that CAS mediates the release of Vpr from the Vpr-NPI-1 complex, thereby allowing its transport into the nucleus. Further investigation of the molecular mechanisms underlying the Vpr/NPI-1 interaction and the selective release of full-length Vpr from NPI-1 and its contribution to HIV-1 replication is required to facilitate a better understanding of the HIV-1 nuclear import process.

## Materials and Methods

### Cell culture

Human cervical HeLa and 293T cells were grown in Dulbecco's modified Eagle's medium (DMEM; Invitrogen, Carlsbad, CA) supplemented with 10% heat-inactivated fetal bovine serum (FBS; Sigma-Aldrich, St. Louis, MO) and GlutaMax (GIBCO Industries Inc., Los Angeles, CA).

### Plasmids

The following plasmids have been described previously: the expression vector, pME18Neo, encoding Flag-tagged wild-type Vpr and its control vector, pME18Neo; the expression vector, pCAGGS, encoding Flag-mRFP-Flag-Vpr (mRFP-Vpr) and its control vector, pCAGGS-Flag-mRFP (mRFP); and the GST expression vector, pGEX-6P-3, encoding GST-tagged-Rch1, -Qip1, -NPI-1 and -Impß [6], [33], [50], [51]. The pCAGGS encoding Flag-mRFP-Vpr_N17C74_ (mRFP-N17C74) was constructed as described previously [50]. For the construction of the expression vector pGEX-6P-3, encoding GST-tagged-Vpr (GST-Vpr), a fragment encoding Vpr was amplified by PCR with the following primers: 5′-GGGGATCCGAACAAGCCCCAGAAGACC-3′ and 5′-CCCTCGAGCTAGGATCTACTGGCTCC-3′ from pME18Neo-Flag tagged Vpr [33], [51] and was subcloned into pGEX-6P-3 (GE Health, Buckinghamshire, UK) at the *Bam*HΙ/*Xho*Ι sites. For construction of the expression vector, GST-green fluorescent protein (GFP)-tagged Vpr (GST-GFP-Vpr), a cDNA encoding histidine tag_6_ (His_6_)-tagged-Vpr (Vpr-His), was amplified from pME18Neo-Flag tagged Vpr by PCR with the primers 5′-GGGATCCATGGAACAAGCCCCAGAAGA-3′ and 5′-GCGGCCGCTCAATGATGATGATGATGATGACCGGTCCCGGGGGATCTACT-3′ and subcloned into the pGEX-6P-1-GFP vector at the *Bam*HΙ/*Not*Ι sites. The plasmid GST-Vpr_N17C74_-GFP (GST-N17C74-GFP) has been described previously [5]. To construct the expression vector, pGEX-6P-3-CAS, a cDNA encoding CAS was prepared from pGEX-6P-2-GFP-CAS [9] and ligated into pGEX-6P-3 at the *Bam*HΙ/*Xho*Ι sites. To construct the expression vector, pGEX-6P-3- Q69LRan, a cDNA encoding wild-type Ran was cloned by PCR from a HeLa cDNA library generated using SuperScript II (Invitrogen) according to the manufacturer's instructions. The N-terminal His-tag fused Ran was then amplified by PCR and subcloned into pGEX-6P-3 (GE Healthcare Life Science). Q69LRan was generated using a QuickChange II Site-Directed Mutagenesis kit (Stratagene) according to the manufacturer's instructions. For construction of the truncated Impα isoform expression vectors, the fragments were amplified by PCR and were subcloned into pGEX-6P-3 at the *Bam*HΙ/*Eco*RΙ sites for Rch1 and NPI-1 and the *Bam*HΙ/*Xho*Ι sites for Qip1. The following PCR primers were used: Rch1 1-69, 5′- CCCGGATCCTCCACCAACGAGAATGCTAATACACC-3′ and 5′-CCCGAATTCCTAGTTGCGGTTTTCCTGCAGCGG-3′; 70_–_475, 5′-CCCGGATCCAACCAGGGCACTGTAAATTGG-3′ and 5′-GGGGAATTCCTAAGCTTCAATTTTGTCTAA -3′; 70–402, 5′-CCCGGATCCAACCAGGGCACTGTAAATTGG-3′ and 5′-CCCGAATTCCTAGTTGGTCACGGCCCACACAGCTTCC-3′; 403–529, 5′-GGGGGATCCTATACCAGTGGTGGAACAG-3′ and 5′-GGGGAATTCCTAAAAGTTAAAGGTCCC-3′; NPI-1 1–75, 5′-CCCGGATCCACCACCCCAGGAAAAGAGAAC-3′ and 5′-GGGGAATTCCTACATGTTATTAATCTGAGCCTCATG-3′; 76–405, 5′-CCCGGATCCGAGATGGCACCAGGTGGTGTC-3′ and 5′-CCCGAATTCTCAGGCCCAAGCTGCTTCTTTTCTTG-3′; 76–451, 5′-CCCGGATCCGAGATGGCACCAGGTGGTGTC-3′ and 5′-GGGGAATTCTCACAGGATATTTTCCAAGCC-3′; 406–538, 5′-CCCGGATCCATCACAAATGCAACTTCTGG-3′ and 5′-GGGGAATTCTCAAAGCTGGAAACCTTCCATAG-3′; Qip1 1–67, 5′-CCCGGATCCGCGGACAACGAGAAACT-3′ and 5′-GGGCTCGAGCTATCTATAATCACCATCTATATCAGAG-3′; 68–393, 5′-CCCGGATCCGTGCAAAATACCTCTCTAGAA-3′ and 5′-GGGGCTCGAGCTAGGCCCAAGCAGCTTCTTTTTGAGTGC-3′, 68–439, 5′-CCCGGATCCGTGCAAAATACCTCTCTAGAA-3′ and 5′-GGGGCTCGAGCTACTAATATATTACTTAG-3′; and 394–521, 5′-CCCGGATCCATAAGTAACTTAACAATTAGT-3′ and 5′-GGGCTCGAGCTAAAACTGGAACCCTTCTGTTGGTAC-3′.

### Protein expression and purification

The recombinant GST-tagged Rch1, Qip1, NPI-1, the deletion mutants, GST-GFP, Impß and CAS were expressed in the *Escherichia coli* strain BL21 CodonPlus (DE3)-RIL (Stratagene, La Jolla, CA) and purified using the Glutathione Sepharose 4FF bead system (GSH System, Amersham Biosciences, Piscataway, NJ) as described elsewhere [6], [50]. GST-Vpr and GST-GFP-Vpr were purified as described elsewhere [52]. After expressing these proteins in *E. coli*, cells were lyzed with Lysis Buffer [10 mM Tris-HCl (pH 8.0), 500 mM NaCl, 1% Triton X-100, 5 mM 2-Mercaptoethanol, 10% (w/v) glycerol and protein inhibitor]. GST-Vpr and GST-GFP-Vpr were purified using the GSH system (Amersham Biosciences) and separated on a His-Trap column (GE Health) [5], [52]. GST-N17C74-GFP protein was purified as described previously [5]. Purified GST-Vpr, GST-GFP-Vpr and GST-N17C74-GFP proteins were dialyzed against transport buffer [TB: 20 mM HEPES-KOH [pH 7.3], 110 mM potassium acetate, 2 mM magnesium acetate, 5 mM sodium acetate and 1 mM dithiothreitol (DTT)]. RanQ69L was expressed from pGEX-6P-3 and purified on GSTrap and HisTrap column (GE Healthcare), and after nucleotide exchange for GTP, GST was digested by PreScission Protease and separated on Hi-Trap SP column (GE Healthcare). Activity was confirmed by binding with GST-Importin β.

To express and purify the Flag fusion proteins, 293T cells (5×10^5^ cells) were transfected with 5 mg of the pCAGGS mammlaian expression vector encoding mRFP-Vpr, mRFP-N17C74 or mRFP using FuGene HD Transfection Reagent (Roche Diagnostics, Basel, Switzerland). Two days after transfection, expressed proteins were purified using ANTI-FLAG M2 agarose (Sigma-Aldrich) as described previously [50].

### *In vitro* nuclear import assay

HeLa cells (2 × 10^6^) were seeded on an eight-well coverslip in a 10-cm dish. After 16 to 24 h of culture, HeLa cells were permeabilized by digitonin in TB on ice for 5 min and washed twice with TB as described previously [25]. The permeabilized cells were incubated at room temperature for 1 h or 30 min with 1% bovine serum albumin, GST-GFP-Vpr (for 1 h), GFP-GST (for 1 h) or GST-N17C74-GFP (for 30 min), and transport substrates in a total volume of 10 µl per sample. After incubation, the cells were washed twice with TB and fixed in 3.7% formaldehyde in TB. Samples were examined using confocal laser scanning microscopy (FV 1000; Olympus, Tokyo, Japan) and the nuclear fluorescence intensity was analyzed with MetaMorph software (Molecular Devices Inc., Downingtown, PA). For each condition, the fluorescence intensity per nuclear surface area was quantified for at least 70 nuclei stained with Hoechst 33342 (ImmunoChemistry Technologies LLC., Bloomington, MN).

### Pull-down assay

Glutathione-Sepharose 4FF beads were coupled with GST-Impα isoforms and their mutants in TB for 1 h at 4^o^C and then in 10 mM Tris-HCl (pH 8.0), 50 mM NaCl, 0.05% NP-40 and 1 mM DTT. Vpr proteins purified from 293T cells transfected with pCAGGS encoding mRFP or mRFP-Vpr were incubated with GST-protein conjugated beads for 2 h at 4^o^C. The beads were washed four times with 500 µl washing buffer [10 mM Tris-HCl (pH 8.0), 150 mM NaCl, 0.2% NP-40 and 1 mM DTT] and bound proteins were eluted by incubation with sodium lauryl sulfate (SDS) sample buffer [100 mM sodium phosphate (pH 7.2), 1% SDS, 10% glycerol, 100 mM DTT and 0.001% bromophenol blue] at 98°C for 5 min. Eluted proteins were fractionated by 10% SDS-polyacrylamide gel electrophoresis (PAGE) and detected by Western blotting with anti-Flag M2 monoclonal antibody (MAb) (Sigma-Aldrich).

### Immunoblotting

Cells or proteins were dissolved in SDS sample buffer, heat-denatured and loaded onto 10% SDS polyacrylamide gels. Separated proteins were transferred to a polyvinylidene difluoride membrane (Immobilon; Millipore, Bedford, MA). After treatment with PBST [20 mM Dulbecco's phosphate-buffered saline (PBS) and 0.05% (v/v) Tween 20] containing 5% skim milk at room temperature for 1 h, the blotted membrane was incubated with anti-Flag MAb (M2) (Sigma-Aldrich), anti-CAS polyclonal antibody (CSE1L, Medical & Biological Laboratories Co. Ltd., Nagoya, Japan), or anti-actin polyclonal antibody (Santa Cruz Biotechnology Inc., Santa Cruz, CA) diluted with PBST containing 3% skim milk at room temperature for 2 h or at 4^o^C for 16 to 18 h. The membrane was rinsed with PBST and incubated with horseradish-peroxidase (HRP)-conjugated goat anti-mouse IgG (Zymed Laboratories, San Francisco, CA) for anti-Flag, HRP-goat anti-rabbit IgGs (Zymed Laboratories) for anti-CAS, or HRP-rabbit anti-goat IgG (Zymed Laboratories) for anti-actin. Each antibody was diluted with PBST containing 3% skim milk. After washing with PBST, the bound antibodies were visualized with ECL^TM^ Blotting Detection Reagents (Amersham Biosciences) followed by exposure to X-ray film (Kodak BioMax^TM^ XAR film, Sigma-Aldrich).

### Surface plasmon resonance (SPR) analysis

SPR experiments were performed using the BIAcore 2000 system (GE Health) at room temperature. Impα isoforms and their mutants were coupled directly to the sensor chip (CM5 research grade, GE Health) via standard N-hydroxysuccinimide and N-ethyl-N-(dimethylaminopropyl) carbodiimide activation. To immobilize the proteins, full-length Rch1 [dissolved in 10 mM sodium acetate buffer (pH 5.0)] full-length Qip1 and full-length NPI-1 [dissolved in 10 mM sodium acetate buffer (pH 4.5)], and their mutants [dissolved in 10 mM sodium acetate buffer (pH 4.0)] were injected onto the sensor surface with HBS EP buffer [10 mM Hepes (pH 7.4), 150 mM NaCl, 3 mM ethylenediaminetetraacetic acid, and 0.05% surfactant P20; GE Healthcare] employed as the mobile phase buffer during the immobilization process. Following immobilization, 50 mM Tris-HCl buffer (pH 7.5) was injected to quench the unreacted N-hydroxysuccinimide groups, and then PBS was used as the mobile phase buffer. GST and GST-Vpr samples at various concentrations were injected as analytes, and bound analytes were subsequently removed by washing with the mobile phase buffer at 300 s after the injection. Vpr sensorgrams were obtained by subtracting GST curves from GST-Vpr curves. Kinetic constants were calculated from the Vpr sensorgrams using the BIA evaluation software, version 3.0 Biacore AB (GE Healthcare). Dissociation constants (K_D_) were calculated from the resonance unit at equilibrium using the following equation: 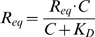
where R_eq_ is the steady state binding level, K_D_ is the dissociation constant and C is the analyte concentration. R_eq_ is related to concentration according to this equation.

### Small interfering RNAs (siRNA)

The siRNAs against CAS were designed with the BLOCK-iT RNAi Designer (Invitrogen). The siRNA forward sequences targeting CAS were 5′-AGCAACAGUGGAUAAUUCUGAUUUC-3′ for siRNA1 and 5′-UUAACUGCUUCUGAAUUUGCUCUGG-3′ for siRNA2. HeLa cells (1 × 10^6^) were seeded on a 6-cm dish. After cells had adhered to the dish, the cells were transfected with the siRNAs using Lipofectamine RNAiMAX (Invitrogen) according to the manufacturer's protocols. After 16 h, cells (2×10^6^) were seeded onto an eight-well coverslip within a 10-cm dish and were used in an *in vitro* import assay following 36 h incubation with an siRNA.

### Statistical methodology

Statistical analyses were conducted using R version 2.8 (1).

## Supporting Information

Figure S1 **Immunofluorescent staining of endogenous CAS in semi-intact cells.** The two panels show the steps involved in cell preparation for the *in vitro* import assay: intact cells (left panel), digitonin-treated cells and the cells incubated on ice for 5 min following digitonin treatment (right panel). Cells on cover slips were fixed with 3.7% formaldehyde in PBS for 15 min at room temperature and permeabilized with PBS containing 0.5% Triton X-100 for 7 min on ice. The cells on the coverslips were incubated with either anti-CAS polyclonal antibody (Green) or anti-Rch1 MAb (Red) in PBS containing 5% skim milk for 1 h at RT. After rinsing with PBS, the cells were incubated with either Alexa-488–conjugated anti-rabbit IgG (for CAS) or Alexa-546–conjugated anti-mouse IgG (for Rch1) antibodies (Invitrogen), or Hoechst 33342 (ImmunoChemistry Technologies LLC.) in PBS containing 5% skim milk for 30 min. After rinsing with PBS, the cover slips were mounted on glass slides in PBS containing 90% glycerol before analysis by confocal laser scanning microscopy. Bar = 10 µm.
(TIF)
