# Supplementary figures and images for: Nuclear Exportin Receptor CAS Regulates the NPI-1–Mediated Nuclear Import of HIV-1 Vpr
Source: PLoS One. 2011 Nov 16;6(11):e27815. doi: 10.1371/journal.pone.0027815 (PMC3218035; doi:10.1371/journal.pone.0027815)

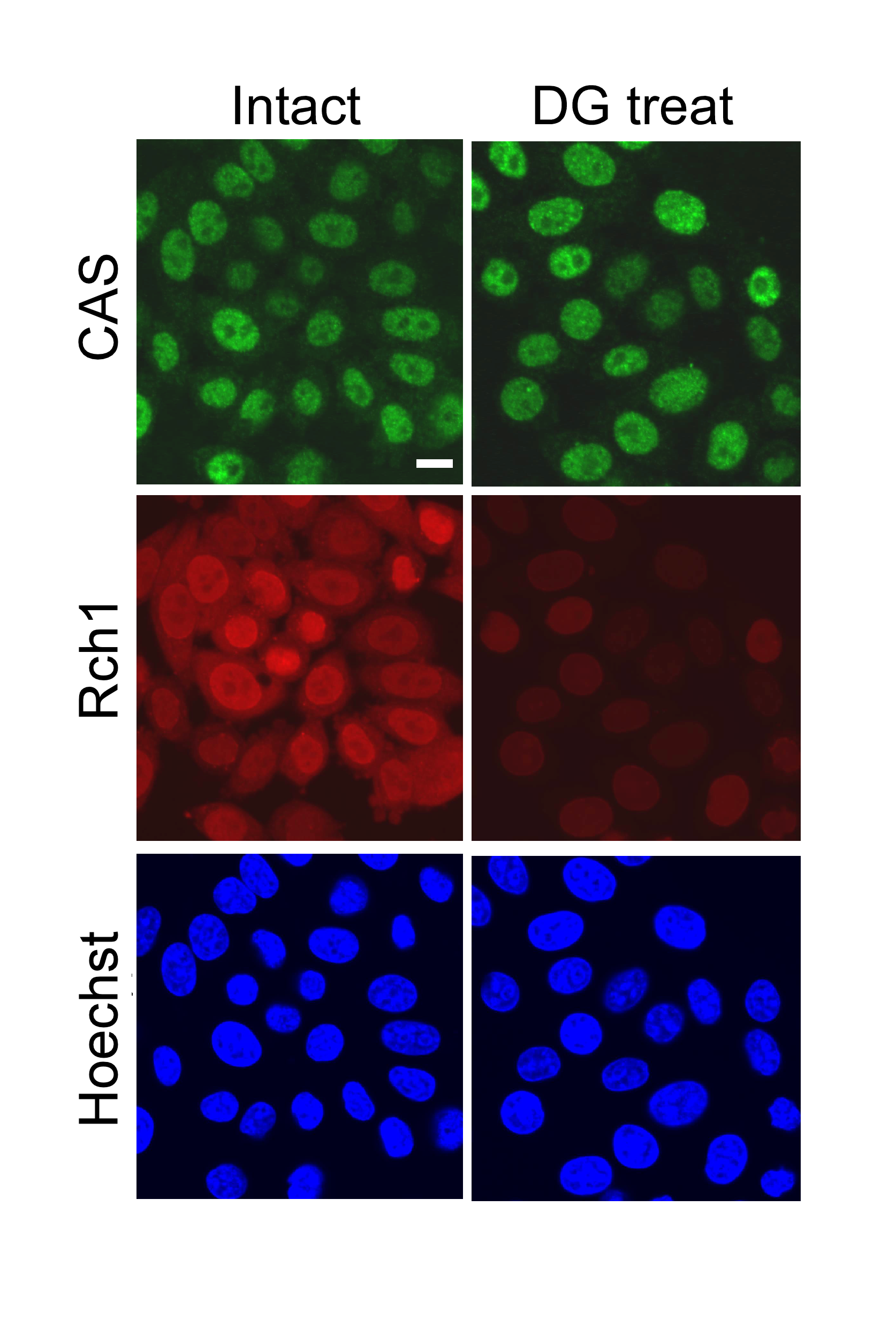

Supplement: Figure S1 — Immunofluorescent staining of endogenous CAS in semi-intact cells. The two panels show the steps involved in cell preparation for the in vitro import assay: intact cells (left panel), digitonin-treated cells and the cells incubated on ice for 5 min following digitonin treatment (right panel). Cells on cover slips were fixed with 3.7% formaldehyde in PBS for 15 min at room temperature and permeabilized with PBS containing 0.5% Triton X-100 for 7 min on ice. The cells on the coverslips were incubated with either anti-CAS polyclonal antibody (Green) or anti-Rch1 MAb (Red) in PBS containing 5% skim milk for 1 h at RT. After rinsing with PBS, the cells were incubated with either Alexa-488–conjugated anti-rabbit IgG (for CAS) or Alexa-546–conjugated anti-mouse IgG (for Rch1) antibodies (Invitrogen), or Hoechst 33342 (ImmunoChemistry Technologies LLC.) in PBS containing 5% skim milk for 30 min. After rinsing with PBS, the cover slips were mounted on glass slides in PBS containing 90% glycerol before analysis by confocal laser scanning microscopy. Bar = 10 µm. (TIF) [file pone.0027815.s001.tif]
